# Case of Rabies Including Experiments on Rabbits

**Published:** 1899-08

**Authors:** E. M. Ranck

**Affiliations:** Philadelphia, Pa.


### REPORTS OF CASES.

### REPORT OF A CASE OF RABIES, INCLUDING EXPERIMENTS ON RABBITS.

By E. M. Ranck, V.M.D.,

PHILADELPHIA, PA.

Fox-terrier dog, two-and-a-half or three years of age, in West Philadelphia:

*History.* At first none that would lead one to suspect rabies, but on examination, after muzzling the animal, found some scars on legs and throat, and on arrival of the man of the house could get history of the animal fighting with another dog about two weeks previous.

*Symptoms.* Unequal dilatation of pupils of eyes, partial paralysis of lower jaw, tongue swollen, staggering gait; when stimulated would show distinct opisthotonus; no bark, but attempts at same, which resulted in a hoarse cough.

*Treatment.* Condemned animal to death, but owing to the fact of a medical student living in the house, and wishing him to have an opportunity to see it, I muzzled the animal securely and isolated him in a well-protected place, and gave him one grain of morphine sulphate hypodermatically. A few hours later chloroformed the animal, and had him sent to me at the laboratory, when a necropsy was made.

*Necropsy.* Some dirt in crevices around teeth and under tongue, throat inflamed and œsophagus contained some dirt mixed with saliva. Stomach contents normal, except one or two small stones about the size of hazelnuts, but no other foreign bodies. Intestines normal, as well as surrounding glands. Bladder well filled, but no evidence of cystitis, and the animal during life had no trouble during micturition. Brain and meninges inflamed, but no one local area could be found to be more intensely inflamed than another, but seemed to be general. Spinal cord not examined farther back than the cervical region.

*Experiment.* After extracting the brain under all the antiseptic precautions possible, macerated small amounts of the fourth ventricle of brain in distilled water; trephined two large rabbits for inoculation. Injected about three-quarters to one c.c.

of the material in each brain cavity, closed the opening, and waited developments. Also inoculated six guinea-pigs by injecting directly into spinal cord near base of brain.

*Results.* Rabbits: One became paralyzed on the twenty-second day and one on the twenty-third day after inoculation. Both died about two days after paralysis. Guinea-pigs: Two died immediately, having pithed them in doing the injecting; two survived and remained perfectly well, due, perhaps, to the fact of not getting material in the proper place; one died fifteen days and one sixteen days after inoculation, and both were paralyzed before they died.

*Conclusion.* From above facts I cannot see where any one can question the prevalence of rabies, for if it is not rabies, what is it? At this writing I have under experiment some brains of dogs which are being passed through rabbits, and the remainder hardened to be studied carefully by bacteriological and pathological experts in the hope of discovering the micro-organism of the disease. Have also had some satisfactory results for other practitioners which they will report later.

